# Network clustering algorithms and preprocessing pipelines for robust cell type identification in single-cell RNA sequencing data

**DOI:** 10.1038/s41598-026-49033-w

**Published:** 2026-05-15

**Authors:** Fatemeh Sadat Fatemi Nasrollahi, Filipi Nascimento Silva, Shiwei Liu, Soumilee Chaudhuri, Meichen Yu, Juexin Wang, Kwangsik Nho, Andrew J. Saykin, David A. Bennett, Olaf Sporns, Santo Fortunato

**Affiliations:** 1 Luddy School of Informatics, Computing, and Engineering, Indiana University, Bloomington, IN USA; 2 Center for Neuroimaging and the Indiana Alzheimer’s Disease Research Center, Indiana University, IN, USA; 3 Luddy School of Informatics, Computing, and Engineering, Indiana University, Indianapolis, IN USA; 4 Rush Alzheimer’s Disease Center (Drs. Bennett, Schneider, and Wilson) and Rush Institute for Healthy Aging (Drs. Bienias and Evans), Rush University Medical Center, Chicago, IL USA; 5 Department of Psychology, Indiana University, IN, USA

**Keywords:** Biological techniques, Computational biology and bioinformatics

## Abstract

Single cell RNA-seq (scRNA-seq) technologies provide unprecedented resolution representing transcriptomics at the level of single cell. One of the biggest challenges in scRNA-seq data analysis is the cell type annotation, which is usually inferred by cell separation approaches. In-silico algorithms that accurately identify individual cell types in ongoing single-cell sequencing studies are crucial for unlocking cellular heterogeneity and understanding the biological basis of diseases. In this study, we focus on robustly identifying cell types in single-cell RNA sequencing data; we conduct a comparative analysis using methods established in biology, like Seurat, Leiden, and WGCNA, as well as network-based methods Infomap, statistical inference via Stochastic Block Models (SBM), and single-cell Graph Neural Networks (scGNN). We also analyze preprocessing pipelines to identify and optimize key components in the process, explicitly considering their role in mitigating inherent data noise and potential batch effects for robust cell type identification. Leveraging three independent datasets, PBMC, ROSMAP, and MOp, we employ clustering algorithms on cell-cell networks derived from gene expression data. Our findings reveal that clusters identified by multiresolution Infomap and Leiden show a closer alignment, with Infomap standing out as a particularly effective approach. Infomap notably offers valuable insights for the precise characterization of cellular landscapes related to neurodegeneration and immunology in scRNA-seq.

## Introduction

In recent years, significant advances in single-cell RNA sequencing (scRNA-Seq) technologies have revolutionized our ability to dissect cellular heterogeneity and uncover deep biological insights^1–5^. Unlike traditional bulk gene expression analysis, which provides average information from a population of cells, scRNA-Seq offers a granular view by quantifying mRNA expression in individual cells. This approach not only facilitates the identification of distinct cell types, it also enables the characterization of cell-specific gene expression profiles and functions. The workflow of single-cell sequencing normally involves isolating individual cells using sophisticated techniques such as Fluorescence-Activated Cell Sorting (FACS) and Droplet-Based Technologies like the 10× Genomics Chromium system. Droplet-based methods have gained popularity for their ability to simultaneously analyze thousands of cells, leveraging Unique Molecular Identifiers (UMIs) to tag the 3$^{\prime }$ end of each transcript and effectively minimize Polymerase Chain Reaction (PCR) bias and batch effect, and accurately annotate transcripts^2,4–6^.

Single-cell transcriptomic studies encompass a wide array of biological and medical inquiries, ranging from delineating cell lineage and identifying novel cell type marker genes to predicting cell fate and exploring gene expression dynamics in disease contexts^7–10^. To achieve these objectives, researchers often employ diverse clustering methods tailored to uncover meaningful patterns within scRNA-Seq datasets^11–14^. These methods streamline the identification of both well-characterized and novel cell types, providing crucial insights into cellular function and behavior under physiological and pathological conditions.

Clustering results vary significantly across different methods, aligning with previous findings^15,16^. Such variation is expected, as clustering methods differ in key aspects, like the concept of cluster itself. Each clustering algorithm has its unique advantages and drawbacks. Therefore, employing multiple clustering methods can provide a comprehensive understanding of cell clusters from different perspectives and a platform to compare the methods. Beyond conventional approaches such as k-means or hierarchical clustering, network-based community detection methods have increasingly been applied to scRNA-seq analysis. These approaches model cells as nodes connected by similarity relationships, allowing the discovery of groups through network topology rather than distance in expression space. Algorithms such as Louvain and Leiden have been widely adopted in popular frameworks like Seurat and SCANPY^17–19^, while other studies have explored stochastic block models and information-theoretic methods such as Infomap^20,21^. Benchmarking efforts have shown that network-based algorithms often provide more stable and biologically meaningful partitions, particularly when the data exhibit complex, nonconvex cluster structures^22,23^.

A major challenge in single-cell RNA-seq (scRNA-seq) analysis lies in selecting optimal clustering parameters, which are crucial for accurately recovering the underlying biological structure of the data. The complexity of this parameter selection process can significantly impact the performance and reliability of the outcomes. Furthermore, scRNA-seq data is inherently susceptible to technical noise (e.g., dropout events, varying sequencing depth) and biological noise, as well as batch effects arising from experimental variations, reagent differences, or diverse patient cohorts. Addressing these factors is paramount for accurate cell type identification. At the same time, it is important that results are robust to parameter choices and remain stable across a broad range of parameter settings. Addressing these challenges necessitates the development of tools that facilitate data analysis and parameter exploration. In particular, interactive visualization interfaces can play a crucial role in enhancing the accessibility and interpretability of clustering analyses in bioinformatics^24^. Such interfaces enable researchers to intuitively explore parameter spaces, assess the stability of clustering results, and make more informed decisions regarding parameter selection.

Previous studies have systematically compared outcomes from multiple methods, including Seurat and Weighted Gene Co-expression Network Analysis (WGCNA)^25^. Many methods predominantly utilize hierarchical clustering and k-means as their clustering strategies^26–28^; however, these techniques have notable limitations. Hierarchical clustering can be computationally demanding, especially with large datasets, and often struggles to identify optimal cluster structures when the data does not naturally follow a hierarchical pattern. Additionally, it normally lacks a clear criterion for selecting meaningful clusters from the many partitions it produces. K-means clustering requires the number of clusters to be specified in advance — an unknown factor in most cases — and assumes clusters are convex in shape, which may misrepresent the true structure of the data.

Therefore, there is a critical need to identify and develop accurate algorithms that surpass such limitations. Certain packages, such as Seurat and SCANPY are widely used for clustering within the field of scRNA-seq data analysis and have considerable potential for further enhancement. Novel algorithms, proposed and refined in other fields, have shown great promise but are often underutilized in scRNA-seq analysis. By systematically evaluating and adapting these cutting-edge algorithms, we can integrate advanced methods into existing scRNA-seq analysis tools and significantly advance the precision and effectiveness of clustering analyses in biological research.

Preprocessing is a crucial step in the analysis pipeline, as it converts raw data into a network representation that can be effectively analyzed by clustering algorithms. This is particularly important in scRNA-seq analysis due to complexity, noise, and high dimensionality of biological data. Effective preprocessing usually consists of multiple steps: normalization to correct for technical variations, noise reduction to minimize random fluctuations, feature selection to highlight the most informative genes, and dimension reduction to simplify the data while preserving key information. These steps are essential for improving data quality and uncovering biological signals. However, preprocessing workflows across different tools are often complex and difficult for users to implement efficiently. Therefore, it is essential to systematically evaluate these preprocessing steps in the context of scRNA-seq data and to comprehensively report their impact on downstream analysis.

In this study, we turn the identification of cell types into a network clustering problem^29–31^. Using gene expression data from distinct scRNA-seq datasets, we construct networks and perform a systematic comparative analysis of state-of-the-art network clustering algorithms, including methods that are seldom applied in biological contexts. While we recognize the value of modern deep learning approaches, our focus here is on network clustering techniques for two main reasons: (1) they are relatively simple and interpretable, and (2) the most effective network clustering algorithms are typically much faster than deep learning methods. Our contribution is twofold: first, to identify the best-performing algorithms for cluster identification, using a reliable ground truth; second, to propose a clear and user-friendly preprocessing pipeline that can be easily tuned for each dataset to achieve optimal results. This work advances the precision and reliability of clustering analyses in biological research. We believe that this study not only contributes to increasing the accuracy of cell type identification, but also provides researchers with a more accessible and effective toolkit for handling complex biological datasets.

## Results

We performed a systematic comparison of six network-based clustering algorithms—Seurat, Leiden, Infomap, WGCNA, Stochastic Block Model (SBM), and scGNN—across multiple single-cell RNA-seq datasets. The analyses were designed to evaluate both algorithmic performance and the influence of preprocessing on clustering stability and biological interpretability. Our study focused on three representative datasets: (1) human Peripheral Blood Mononuclear Cells (PBMCs, 68k cells) generated using the 10× Genomics Chromium platform, (2) the ROSMAP single-nucleus RNA-seq dataset from human brain tissue, and (3) the mouse primary motor cortex (MOp) dataset available through the NeMO Analytics portal.

Each algorithm represents a distinct approach to network community detection. Seurat applies modularity optimization using the Louvain algorithm, while Leiden improves upon Louvain by avoiding that clusters are disconnected, a well-known problem of Louvain. Infomap identifies modules by modeling the flow of information across the network and minimizing its description length; its Markov time parameter determines the temporal scale of diffusion, thereby adjusting cluster resolution. WGCNA constructs weighted co-expression networks and identifies modules via hierarchical clustering, emphasizing correlation structure between cells. The Stochastic Block Model (SBM) infers clusters probabilistically by fitting a generative model to the observed network; the nested SBM formulation yields multiple hierarchical partitions that reveal structure at different resolutions. Finally, scGNN leverages graph neural networks to learn cell embeddings that capture nonlinear transcriptional relationships. Further details on these algorithms and their implementation are provided in Section "Clustering algorithms for scRNA-seq analysis".

For each dataset, cell–cell similarity networks were constructed under both the default Seurat preprocessing pipeline and a set of alternative pipelines that systematically varied normalization, log transformation, feature selection, and dimensionality reduction. Clustering performance was quantified using the Adjusted Rand Index (ARI) when ground truth cell type labels were available, and the Omega index when cells exhibited overlapping marker-gene signatures.

### Results on PBMCs: Seurat-generated networks

**Fig. 1:**
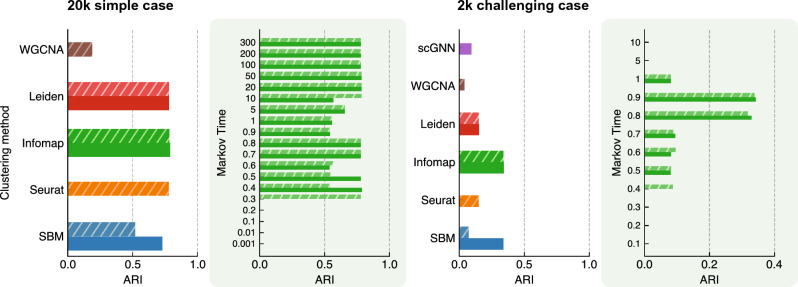
Adjusted Rand Index (ARI) between cell types and detected clusters for SBM, Seurat, Infomap, Leiden, WGCNA, and scGNN in the 20k simple (left panel) and 2k challenging PBMCs datasets (right panel). scGNN is not included in the left panel due to its high time complexity. Both the weighted (hatched) and unweighted (unhatched) versions of the same networks were considered for algorithms that can handle both. The zoomed-in panels in green illustrate the ARI for Infomap across different Markov times.

In this section we present the results of applying the six clustering algorithms described in section "Clustering algorithms for scRNA-seq analysis" to the networks obtained from the PBMCs data (see section “Data sets”). The networks analyzed in this section are generated using the Seurat full pipeline as described in section “Preprocessing techniques”. Clustering analysis can be conducted with or without considering edge weights produced by Seurat, allowing for a comparison of clustering algorithms’ performance in both the weighted and the unweighted case. As described in section “Data sets”, we analyzed three versions of the PBMC dataset: a 20k-cell subset containing distinct immune cell types (simple case), a 2k-cell subset composed of closely related T-cell populations (challenging case), and the full 68k-cell dataset representing all PBMCs. The similarity between the detected clusters and the cell types is estimated via the Adjusted Rand Index (ARI)^32^, which reaches a maximum of 1 when the partitions are identical, and around 0 if they are randomly correlated. First, we consider the 20k dataset, which comprises cells from highly distinct cell types. Figure 1 (left) illustrates the ARI for each method. In this study, the Leiden and Infomap algorithms were executed 100 times each. For Leiden, the partition with the highest modularity was selected, while for Infomap, the partition with the lowest description length was chosen. Due to the higher computational demands of SBM, it was run 20 times, and the partition with the lowest description length was used for the ARI calculation. In contrast, Seurat, WGCNA, and scGNN were each run once, and their respective partitions were directly used to compute the ARI.

Given the well-separated cell type populations in this dataset (see the networks in Fig. 13), high clustering performance is expected across most algorithms. This is supported by the results shown in Fig. 1 (left panel), where Seurat, Infomap, and Leiden each achieve an ARI of 0.78, while SBM attains a slightly lower ARI of 0.73. In contrast, WGCNA performs poorly with an ARI of 0.19, likely due to its reliance on constructing a scale-free network^33^, which may not align well with the characteristics of this dataset. In this context, a KNN-based network (see “Preprocessing techniques”) appears to provide a more effective representation of the data. The green box in Fig. 1 (left panel) further examines Infomap’s performance across varying Markov times, demonstrating its robustness and stability over a broad parameter range — making it the most consistent method in this setting. For a detailed comparison with other algorithms across different resolution parameters, refer to Fig. 14. Note that scGNN was excluded from this analysis due to its high computational cost on this large dataset.

To rigorously evaluate and compare the performance of the algorithms on more challenging cell types — those with similar gene expression profiles — we extracted a representative sample of 2,000 cells. This sample was randomly selected while maintaining the same proportional distribution of cell types as in the original dataset. Figure 1 (right panel) shows the results of this analysis. This dataset presents significant challenges for all clustering methods (see the networks in Fig. 15). The highest ARI achieved is 0.34, for SBM and Infomap. Seurat and Leiden perform less effectively, with an ARI of 0.15. Notably, WGCNA and scGNN exhibit poor performance on this dataset, as illustrated in the figure. The zoomed-in figure in green highlights the same challenge when applying Infomap to this dataset, showing that its performance stability is confined to a narrower range of Markov times. Nevertheless, when compared with other methods across various resolution parameters in Fig. 16, Infomap has a superior performance overall.

**Fig. 2:**
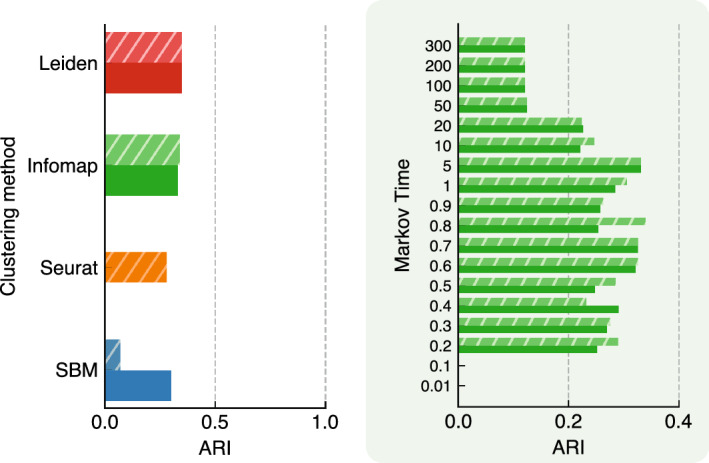
Adjusted Rand Index (ARI) between cell types and detected clusters for SBM, Seurat, Infomap, and Leiden in the 68k PBMCs full dataset. WGCNA and scGNN are not included due to their high time complexity. Both the weighted (hatched) and unweighted (unhatched) versions of the networks were considered for algorithms that can handle both. The zoomed-in figure in green illustrates the ARI for Infomap across different Markov times.

Finally, we applied the algorithms to the complete dataset, encompassing all 68k PBMCs. Figure 2 reports the ARI for the methods that were able to complete the analysis in a reasonable time interval. The ARIs achieved are 0.35, 0.34, 0.30, and 0.27, corresponding to Leiden, Infomap, SBM, and Seurat, respectively. The zoomed-in figure in green highlights the performance stability across a Markov time range of 0.2 to 5. The same stability is not observed for other methods (see Fig. 17). This further confirms that Infomap not only identifies a partition that aligns more closely with the true cell type labels than the other methods, but also maintains stability and robustness across a wide range of Markov times.

### Results on PBMCs: alternative preprocessing

**Fig. 3:**
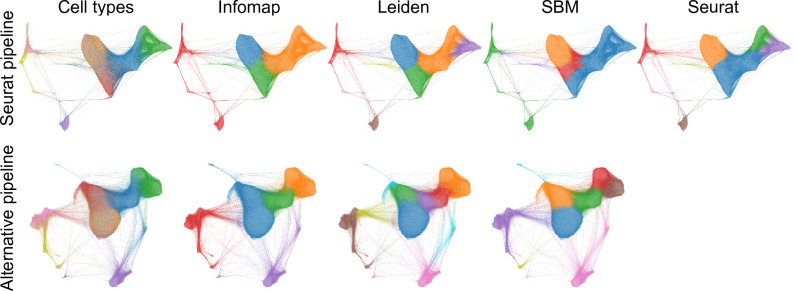
Visualizations of the generated networks from Seurat’s (top row) and alternative (bottom row) pipelines using the full PBMC dataset. The colors correspond to the cell types in the first column on the left, and clusters identified by the clustering techniques otherwise. The visualizations were obtained using the Force Atlas 2 algorithm^34^ implemented in Helios-Web^35^.

**Fig. 4:**
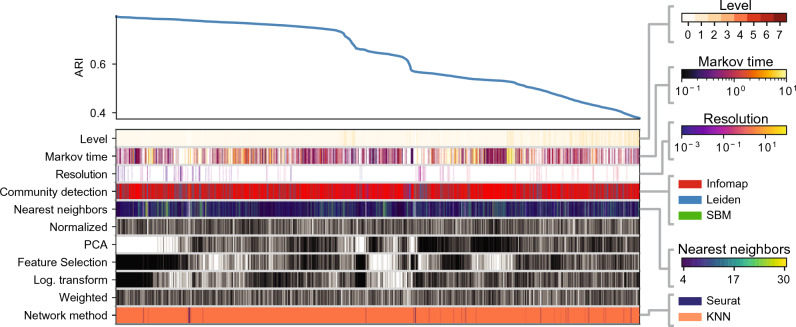
ARI across different configurations of alternative preprocessing pipelines for the 20k PBMC simple case. The top left curve reports the ARI across the top 5000 preprocessing procedures. Each column defines the specific pipeline adopted, by specifying which ingredients are present, according to the color of the blocks. For ingredients “Weighted”, “Log. transformation”, “Feature selection”, “PCA”, and “Normalization” black (white) blocks correspond to having (omitting) that ingredient in the pipeline. Color bars on the right represent the value or category of the rest of the blocks in the columns.

**Fig. 5:**
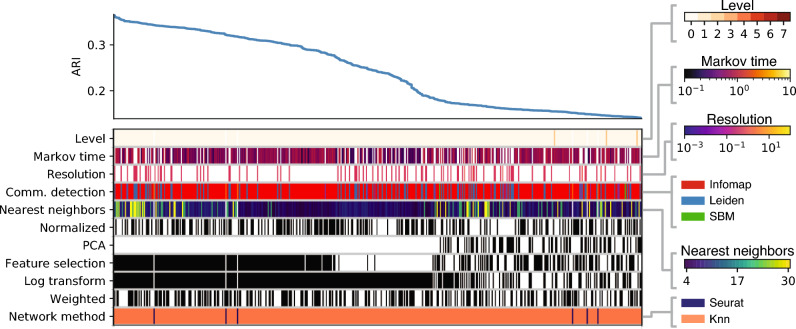
ARI across different configurations of alternative preprocessing pipelines for the 2k PBMC challenging dataset, similar to Fig. 4. The top left curve reports the ARI across the top 500 preprocessing procedures. A version for the top 5000 procedures is available in Figure 18.

**Fig. 6:**
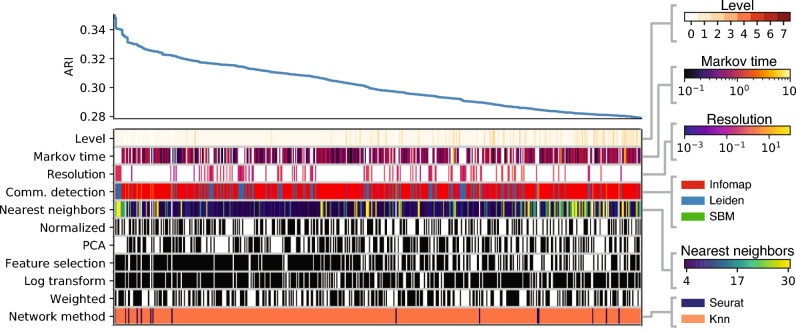
ARI across different configurations of alternative preprocessing pipelines for the 68k PBMC full dataset, similar to Fig. 4. The top left curve reports the ARI across the top 500 preprocessing procedures. A version for the top 5000 procedures is available in Fig. 19.

In this section we explore different combinations of preprocessing steps to generate networks and then apply the clustering algorithms. The aim is to identify pipelines that lead to network representations whose topology makes cell types more visible. We also compare the results acquired from the networks generated by Seurat’s pipeline with the ones obtained from the alternate pipelines we explored. In Fig. 3 we show the clusters found by each clustering algorithm on the network generated from the full 68k dataset by Seurat’s pipeline (top row) and the network generated using the alternative pipeline with the highest correspondence between cell types and detected partitions (bottom row). The alternate pipeline in this case consists of applying KNN with 20 neighbors, normalization, log-transformation, and feature selection. Although the two network structures have a similar overall structure, they exhibit considerable differences. In particular, the community structures vary significantly across the methods and network types, resulting in different Adjusted Rand Indices (ARIs) among them. These ARIs are compared in Figs. 4, 5, and 6 for the three PBMC datasets.

Figure 4 represents the top 5000 preprocessing combinations yielding the highest ARI for the 20k dataset with highly distinct cell types. The top left curve displays the ARI across various configurations. Below it, the columns indicate the combinations of preprocessing steps and clustering algorithms used to achieve each specific ARI, with color bars on the right representing the value or category of each block in the columns. This figure demonstrates that the networks which yield the highest ARIs are generated using a KNN-based pipeline, feature selection, and log transformation, while PCA was not utilized. Additionally, the highest ARI values for the alternate pipeline exceeded 0.795 — outperforming the results achieved using Seurat’s pipeline described in Section "Results on PBMCs: Seurat-generated networks"; KNN with a relatively small number of neighbors exhibits exceptional performance. The number of possible configurations within Seurat’s analytical pipeline is notably smaller compared to the KNN-based approach. This distinction arises because the analysis in section "Results on PBMCs: Seurat-generated networks" is limited to variations in community detection algorithms and their associated parameters. This disparity is evident in the bottom row of Figure 4, where the dominance of the KNN-based pipeline can be attributed to the substantially greater number of combinatorial possibilities available in the KNN-based approach compared to the Seurat pipeline. Consistent with the findings presented in Section "Results on PBMCs: Seurat-generated networks", Infomap and Leiden outperform the other clustering methods. These results also indicate that certain choices in the pipeline such as using weighted versus unweighted edges and the normalization step, do not significantly affect the outcome. This observation is data-specific and does not necessarily generalize to other datasets.

Figure 5 shows the ARIs across pipelines in the same settings for the challenging 2k sample of the PBMC 68k dataset. Similar to the previous case, the best results were obtained when feature selection and log transformation were applied, while PCA was not. The use of weights or normalization has no significant impact on performance. In this instance, however, KNN with a higher number of neighbors ($>15$) resulted in the highest ARI. Infomap emerges as the highest-performing clustering method overall. However, in certain cases, Leiden surpasses Infomap; Given that their ARI values are closely comparable, and Leiden is substantially more computationally efficient, Leiden may be a preferable choice—depending on the specific objectives and constraints of the clustering analysis.

Finally, Fig. 6 presents the results of the same analysis for the full PBMC 68k dataset. Notably, Seurat’s pipeline has the highest overall performance. However, within the top 5000 scores (Fig. 19), we found similar scores for pipelines using the KNN representation and various combinations of preprocessing steps. Interestingly, neither PCA nor normalization play a role in the pipeline. In contrast, feature selection and log transformation appear to play a crucial role in the performance on this dataset. These observations suggest that for the full PBMC 68k dataset — and consistently across its subsets — the most effective pipelines are those that utilize a KNN graph representation combined with feature selection and log transformation. This configuration yields the highest clustering accuracy.

The limited impact of PCA and normalization on performance in this setting may be attributed to the intrinsic properties of the dataset. The PBMC 68k dataset contains a large number of well-separated cell populations, which may reduce the need for dimensionality reduction via PCA. Moreover, since log transformation already stabilizes variance and mitigates the influence of highly expressed genes, additional normalization steps may provide minimal added value. As a result, the combination of feature selection and log transformation likely preserves the most biologically informative structure while maintaining computational efficiency.

### Results on ROSMAP: Seurat-generated networks

In this section, we present the results of applying the clustering algorithms described in section “Preprocessing techniques” to networks derived from the ROSMAP dataset, as detailed in section “Data sets”. We employed the preprocessing pipeline from Seurat, following the same approach outlined in section "Results on PBMCs: Seurat-generated networks". While the dotplot in Fig. 11 shows close alignment between marker gene expression levels and the assigned cell types, the latter is inferred through computational steps. A more concrete approach to evaluate the clustering results is to use the marker gene expression data as the ground truth; in this section, we use them as discriminants. Ideally, cells are considered to belong to the same type if they all express the same marker genes. Therefore we consider the marker genes as cluster labels. However, since the same cell can express multiple marker genes, we end up with a so-called fuzzy partition as ground truth here, where each cell has multiple labels. Fortunately, the literature provides several metrics for quantifying the similarity between fuzzy partitions and the hard partitions produced by clustering algorithms. A popular metric is the Omega index^36^(see Appendix for the mathematical details). The Omega index ranges between 0 and 1; a value closer to 1 indicates a higher agreement between the two partitions. In this dataset, cell types “OPC” and “oligodendrocytes” exhibit highly similar gene expression profiles. Cells labeled with “neuron”, “excitatory neuron” and “inhibitory neuron” also share substantial similarity in gene expression. Such overlaps introduce significant fuzziness in the ground truth, and we anticipate that the Omega index will not be particularly high across methods. Nonetheless, the goal is to identify which clustering algorithm provides the highest and most robust Omega index values, reflecting the best performance. We calculated the Omega index using the Python package developed by Murray et al.^37^.

**Fig. 7:**
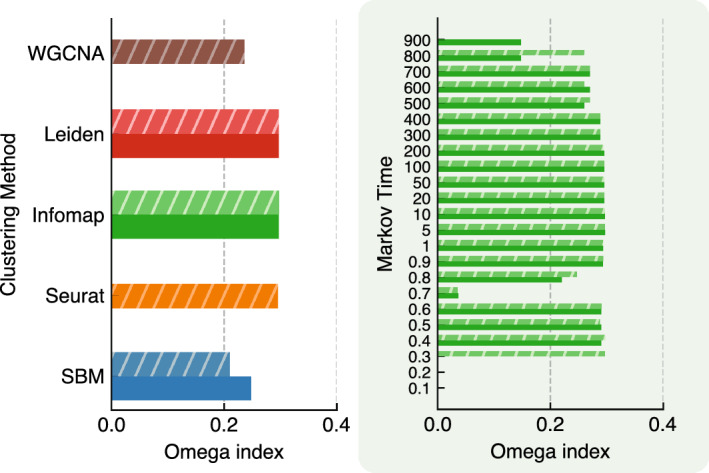
Omega index between ground truth and detected clusters for SBM, Seurat, Infomap, Leiden, and WGCNA in the full ROSMAP dataset. Both the weighted (hatched) and unweighted (unhatched) versions of the same networks were considered for algorithms that can handle both. The zoomed-in panel illustrates the Omega index for Infomap across different Markov times.

The results illustrated in Fig. 7 show the Omega index achieved by each clustering method. Notably, the highest scores are attained by Infomap, Leiden, and Seurat, reaching a value of 0.29. Moreover, the zoomed-in figure in green showcases the range of Markov times (0.3 to 800) over which Infomap’s performance is stable, thus reinforcing the conclusions drawn from the analysis of PBMC data. For comparisons with other methods across different resolution parameters, see Fig. 20. The SBM and WGCNA methods yield Omega indices of 0.25 and 0.23, respectively, reflecting a slight decrease in performance. However, their substantial time complexity poses a significant challenge. Leiden, Seurat, and Infomap exhibit considerably lower time complexity, thereby showing their superiority in practical applications.

The cell type annotations yield the same Omega index values as those obtained from the partitions identified by three clustering algorithms, indicating a high level of agreement and supporting the reliability of these clustering results. Indeed, in Fig. 21 we reported the ARIs between such cell types and the partitions obtained from the clustering algorithms. Figure 21 shows the same trends observed in Fig. 7. Notably, the highest ARIs are attained by Infomap, Leiden, and Seurat, reaching a value of 0.96 (see Fig. 22 for the illustration of the network). This confirms the strong alignment between the assigned cell types and the clusters found by the algorithms. Also, Infomap’s performance is stable, thus reinforcing the conclusions drawn from the prior analyses. For a comparison with other methods across different resolution parameters, see Fig. 23.

### Results on ROSMAP: alternative preprocessing

**Fig. 8:**
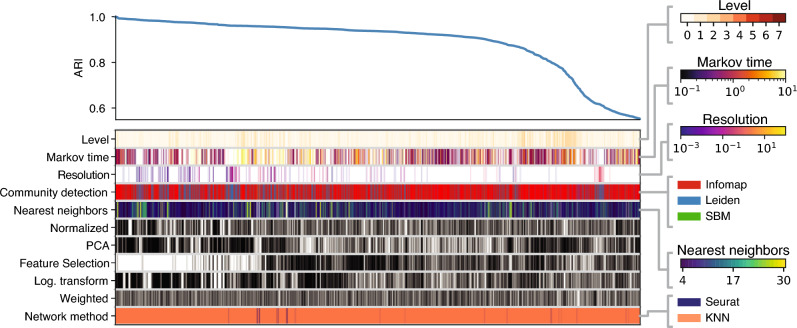
ARI across different configurations of alternative preprocessing pipelines for the ROSMAP dataset, similar to Fig. 4. The top left curve reports the ARI across the top 5000 preprocessing procedures.

In this section, we evaluate a wide range of preprocessing pipelines for the ROSMAP dataset by systematically combining different preprocessing steps to construct networks, followed by clustering using various algorithms. The experimental setting mirrors that of Section "Results on PBMCs: Alternative preprocessing". Figure 8 displays the top 5000 preprocessing combinations ranked by Adjusted Rand Index (ARI).

Notably, the highest ARI scores are consistently achieved using pipelines that include log transformation, PCA, and normalization, while feature selection appears to have a detrimental effect on clustering performance in this context. Additionally, Infomap once again emerges as the top-performing clustering algorithm across most configurations. These observations are consistent with our earlier findings and demonstrate Infomap’s robustness across diverse datasets and preprocessing schemes.

One possible explanation for the reduced effectiveness of feature selection in this dataset is that it may inadvertently exclude genes that, while individually weakly informative, collectively contribute to meaningful network structure—especially in complex and heterogeneous brain tissue such as that from ROSMAP. In contrast, PCA and normalization may help reduce technical noise and batch effects (e.g., from different donors), allowing the network to better capture underlying biological signals. The strong performance of log transformation is also expected, as it helps stabilize variance and down-weight extreme gene expression values, making the resulting similarity structure more reflective of meaningful cell–cell relationships. These results highlight the importance of tailoring preprocessing strategies to the characteristics of the dataset and further demonstrate that appropriate preprocessing is crucial for achieving reliable clustering outcomes in scRNA-seq data analysis.

### Results on MOp: Seurat-generated networks

**Fig. 9:**
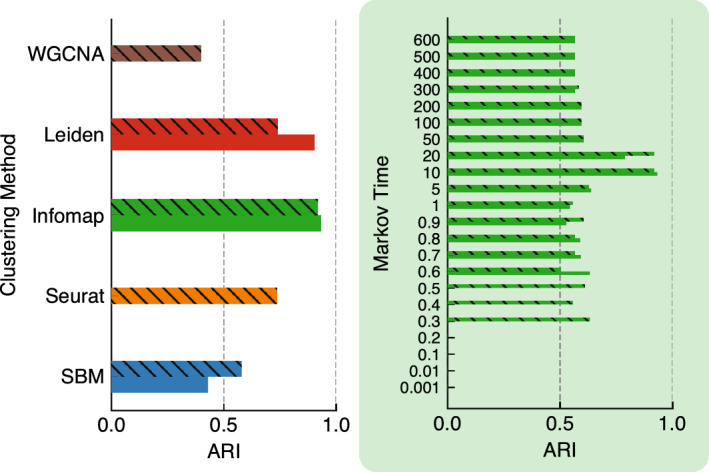
Adjusted Rand Index (ARI) between cell types and detected clusters for SBM, Seurat, Infomap, Leiden, and WGCNA in MOp 10k dataset. Both the weighted (hatched) and unweighted (unhatched) versions of the same networks were considered for algorithms that can handle both. The zoomed-in panels in green illustrate the ARI for Infomap across different Markov times.

In this section, we present the results of applying the clustering algorithms to the networks obtained from the MOp dataset (see Section “Data sets”). The networks analyzed here were generated using the complete Seurat pipeline. In this dataset, the cell types are relatively well separated as illustrated in Fig. 24, so we expect all clustering methods to achieve comparatively high ARI values. Indeed, our findings from the MOp dataset in Fig. 9 are consistent with the earlier observations made on the PBMC and ROSMAP datasets, showing a similar relative ranking of algorithmic performance. Among the evaluated methods, the unweighted Infomap algorithm achieves the highest ARI of 0.93, indicating highly accurate recovery of the reference cell type labels. The Leiden algorithm follows closely with an ARI of 0.90, confirming its robustness and effectiveness across distinct datasets. In contrast, WGCNA and SBM show markedly lower performance, underscoring their limited ability to delineate well-defined transcriptional clusters in this context. As highlighted by the green zoom-in panel, the Infomap results also demonstrate notable stability across a wide range of Markov times—maintaining ARI values above 0.5 even under substantial parameter variation.

### Results on MOp: alternative preprocessing

**Fig. 10:**
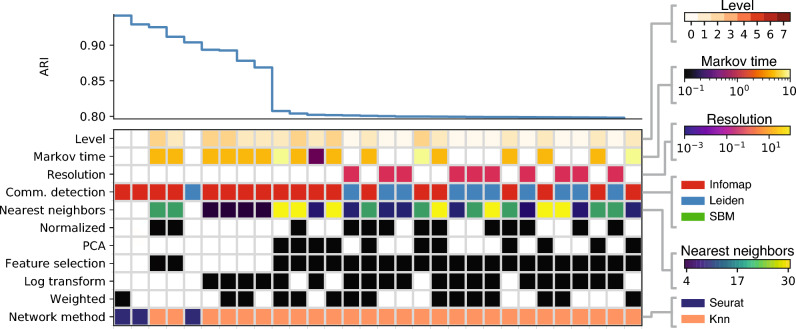
ARI across different configurations of alternative preprocessing pipelines for the MOp 10k dataset, similar to Fig. 4. The top left curve reports the ARI across the top 50 preprocessing procedures. A version for the top 5000 procedures is available in Figure 26.

Figure 10 reports the ARI values obtained from different combinations of preprocessing steps used to construct the cell–cell networks, followed by clustering. The ARI scores range from approximately 0.80 to 0.95, indicating that this dataset presents a comparatively easier clustering problem. As observed in previous analyses, PCA does not improve performance. The networks generated using Seurat achieve one of the top two ARI values, but only when Infomap is applied. Whether edge weights are included appears to have minimal impact, and the same holds for log transformation and feature selection.

## Discussion

Single-cell RNA sequencing (scRNA-seq) has emerged as a critical technology for dissecting cellular identity and function at high resolution, providing unprecedented insight into complex tissues like the brain. In neurodegenerative disease research, scRNA-seq enables the identification of diverse cell types and states and reveals molecular mechanisms that drive pathology^38^. Clustering such single-cell data into biologically meaningful groups is essential for interpreting cell-type heterogeneity and dynamics. Network-based clustering approaches have become a standard in scRNA-seq analysis, and there is growing interest in systematically comparing their performance across diverse datasets. While several benchmarking studies have evaluated clustering algorithms, there is still a need for more comprehensive comparisons that include both classical methods and some of the newer network-based approaches. Previous studies have compared clustering algorithms for single-cell RNA-seq analysis, highlighting substantial variability in performance and sensitivity to preprocessing and parameter choices^15,22,23,39^. These benchmarks have provided valuable insights into the relative strengths of widely used frameworks such as Seurat, SCANPY, and SC3, and have emphasized that no single method performs optimally across all datasets or parameter settings. Building on this foundation, our study focuses specifically on network-based community detection methods—including Leiden, Infomap, and the Stochastic Block Model—and evaluates how alternative preprocessing pipelines influence their performance. By including these network-based methods under consistent experimental settings, we provide a unified assessment of their resolution-dependent behavior and robustness—an analysis not undertaken in previous benchmarking studies.

Leiden is an improved modularity optimization algorithm derived from the Louvain method and is widely used in single-cell analyses due to its speed and ability to uncover well-connected communities. Infomap is an information-theoretic community detection algorithm that optimizes the map equation; it identifies clusters by modeling information flow on the network. We included an SBM inferential approach as a model-based graph clustering strategy – SBMs provide a principled probabilistic framework for community detection, and recent work has proposed SBMs as a viable alternative for single-cell data clustering^21^.

We also implemented WGCNA, a method designed for weighted gene correlation network analysis, widely used for identifying gene modules and inferring regulatory networks. Additionally, we included Seurat, a widely adopted framework that integrates preprocessing, dimensionality reduction, and clustering, including Louvain modularity maximization, ensuring our analysis aligns with standard bioinformatics practices. Finally, we incorporated scGNN, a graph neural network-based approach that leverages deep learning for cell–cell relationship modeling, adding a complementary perspective to our evaluation of clustering methods.

Recent advances in graph neural networks have extended single-cell analysis into the multi-omics domain^40,41^. These approaches demonstrate the power of GNNs for capturing complex cross-layer dependencies; however, they differ fundamentally from the single-layer setting assessed in our work. Our benchmarking focuses on the clustering performance of graph-based algorithms operating on single-modality scRNA-seq data, providing a controlled comparison that complements these multi-omics GNN studies. In parallel, other recent developments such as HiCat^42^ employ semi-supervised or reference-based classification to improve automated cell annotation. While these methods achieve strong performance when reliable reference labels are available, they address a distinct task from unsupervised network-based clustering, which aims to discover cell populations without prior annotation.

Furthermore, our study underscores the importance of preprocessing, as it directly influences clustering outcomes in single-cell RNA-seq analysis. To systematically evaluate its impact, we explored multiple preprocessing pipelines while adjusting parameters for different clustering algorithms. Our results suggest that KNN-based graph construction plays a crucial role in achieving optimal clustering performance, whereas commonly used steps like log-normalization, PCA, and feature selection may not always be necessary, depending on the dataset and its inherent noise characteristics. These findings align with prior studies highlighting the variability in preprocessing effects across different analytical frameworks^43^. While widely adopted pipelines such as Seurat include these steps by default, our analysis suggests that their relevance may depend on the properties of the data, particularly concerning the presence and nature of noise and batch effects. By providing a systematic evaluation of preprocessing strategies, we aim to offer practical insights that may assist researchers in optimizing their workflows for single-cell clustering.

An important factor in the success of graph-based clustering methods is the availability of efficient and well-integrated single-cell analysis frameworks. SCANPY^19^ and Seurat both provide streamlined pipelines that encompass key preprocessing steps such as data normalization, dimensionality reduction, nearest-neighbor graph construction, and clustering, including Leiden and Louvain as widely used options. These frameworks offer optimized implementations that allow researchers to perform large-scale single-cell clustering with minimal computational overhead while ensuring reproducibility across studies. Given Infomap’s strong performance in both accuracy and runtime, an important extension to these frameworks would be to incorporate Infomap as an alternative clustering method. Since Infomap provides a different methodological approach — optimizing information flow rather than modularity — it could complement existing tools by capturing distinct network structures that Leiden might overlook. By integrating Infomap into SCANPY and Seurat, these widely adopted frameworks could offer users an additional, computationally efficient, and biologically meaningful clustering strategy, further enhancing single-cell data analysis.

We focused our analysis on the PBMC, ROSMAP, MOp datasets. PBMC and MOp provide cell annotations based on biological knowledge rather than computational inference. For ROSMAP, although some level of computational annotation is involved, we filtered the data to retain only cells with well-supported, confidently assigned labels. We acknowledge that relying on only three datasets is a limitation; however, this type of analysis — evaluating the correspondence between computational clustering and biological annotation — requires high-quality ground truth, and PBMC and MOp were the only datasets we could identify with annotations derived purely from biology.

## Methods

### Clustering algorithms for scRNA-seq analysis

This section presents a concise review of the network clustering algorithms applied in this study. As inherently unsupervised approaches, these methods identify clusters — cells with similar expression patterns — without relying on ground truth labels. These include Leiden^44^, Infomap^20,45^, WGCNA^46^, statistical inference through the Stochastic Block Model (SBM)^47,48^, Seurat^17,18^, and a deep learning based model - single-cell Graph Neural Network (scGNN)^49^.

**Leiden** optimizes modularity, a quality function that rewards partitions with higher density of edges within clusters than expected in a randomized network with the same degree sequence of the input one^44,50^. The resolution parameter in the Leiden algorithm allows one to adjust the level of granularity of the analysis, going from small to large clusters, as required by the biological context of the dataset. Moreover, Leiden can operate on both weighted and unweighted networks. Particularly with the incorporation of edge weights, the Leiden algorithm can detect cell clusters with nuanced transcriptional variances.

**Infomap** is designed to identify clusters in networks by optimizing the compression of the information used to describe a diffusion process running on the network^45^. Infomap includes a parameter known as the Markov time^51^, which governs the pace of the exploration of the network’s structure via the diffusion process and influences the granularity of clustering. Similarly to changing the resolution parameter in Leiden, adjusting the Markov time provides a mechanism to fine-tune the clustering resolution according to the desired level of detail in the analysis. Infomap is capable of analyzing both weighted and unweighted networks.

**WGCNA (Weighted Gene Co-expression Network Analysis)** analyzes correlation patterns in gene expression data across multiple samples. Typically, it constructs a weighted gene co-expression network to identify modules of highly correlated genes associated with specific biological functions or traits using hierarchical clustering^46,52^. WGCNA is widely used in the analysis of regulatory networks and gene co-expression relationships in complex biological systems. In this work, we adapted WGCNA to cluster cells instead of genes. By analyzing gene expression profiles across cells, WGCNA constructs a cell co-expression network and identifies clusters of cells with similar expression patterns. In this study, due to the considerable size of the gene space, the conventional approach of constructing a cell co-expression network directly encountered significant challenges. To address this, Principal Component Analysis (PCA) was employed to effectively reduce the dimensionality of the dataset. Specifically, we use 50 principal components in the study, which is the default setting in Seurat, to maintain consistency.

**Statistical inference via Stochastic Block Model (SBM)**. The Stochastic Block Model is a probabilistic generative model of networks with community structure^53^. Its distinctive feature is that the probability that two nodes are connected solely depends on their community memberships. By fitting an SBM to a network, one then recovers the partition upon which the model generates a network most similar to the input one. Nested SBM derives a hierarchical partition, with clusters including (included in) smaller (larger) clusters^47,54^. Such a hierarchical approach facilitates the identification of cluster structures at different resolutions, as the different levels of the hierarchy correspond to different cluster sizes.

**Seurat** provides built-in functions for clustering scRNA-seq data^17,18^. These functions enable the partitioning of cells into clusters based on gene expression similarity or network connectivity. Seurat uses Louvain modularity Optimizer by^55^ as the default modularity maximization method. Since the Leiden algorithm is just a modification of Louvain, the clustering outcomes from Seurat are expected to exhibit agreement with the partitions derived from Leiden. Throughout this work, the term “Seurat” specifically refers to the Seurat framework utilizing the Louvain modularity maximization algorithm using the function FindClusters().

**scGNN (single-cell Graph Neural Network)** stacks graph neural networks in an iterative framework tailored for scRNA-seq analysis^49^. It leverages graph neural networks to capture complex relationships in cell similarity networks. scGNN learns low-dimensional embeddings for cells, facilitating clustering and imputation tasks while preserving biological relevance. scGNN considers unweighted interactions in the construction of these networks.

### Data sets

We used a well-characterized 68k human Peripheral Blood Mononuclear Cells (PBMCs) scRNA-seq dataset via the 10× Genomics Chromium platform^4,5,56^. The PBMC dataset comprises single-cell transcriptomes from 68k freshly isolated PBMCs. PBMCs represent a heterogeneous population of white blood cells in peripheral blood, consisting of mostly lymphocytes (T cells, B cells, and natural killer (NK) cells), monocytes, and dendritic cells. Cell types were assigned by first down-sampling each purified PBMC population to 16,000 confidently mapped reads per cell. Then, the average gene expression profile was calculated for each population. The gene expression of each cell in the complex population was compared to these profiles using Spearman’s correlation. Each cell was assigned the ID of the purified population with which it had the highest correlation. These annotation and preprocessing steps were carefully performed by the original authors, and we used the published dataset together with their validated cell-type labels for all analyses in this study. The PBMC dataset is notable for its balance between cell count and the features provided by droplet-based technology. The diverse array of profiled cells supports a detailed characterization of immune cell diversity, enabling the detection of less abundant cell populations that might be challenging to identify in smaller datasets. Similarly to the works of Sun et al^57^. and Huh et al^25^., we sampled the data as follows:

1. 20k simple case: This subset comprises all 20k cells from three distinctly discernible cell types: CD56+NK cells, CD19+B cells, and CD4+/CD25+ regulatory T cells.
2. 2k challenging case: This subset includes 2k cells from three closely related cell types with similar expression profiles: CD8+/CD45RA+ naive cytotoxic T cells, CD4+/CD25+ regulatory T cells, and CD4+/CD45RA+/CD25- naive T cells.

Each of these subsets, along with the full dataset of 68k PBMCs, was used as a separate input to conduct a comparative analysis of the clustering algorithms’ performance across different subsets.

Furthermore, we utilized the ROSMAP single-cell RNA sequencing dataset, derived from the dorsolateral prefrontal cortex (DLPFC) region of 12 male and 12 female participants, as described by Cain et al.^58^. ROSMAP stands for Religious Orders Study (ROS)/Rush Memory and Aging Project (MAP); ROSMAP enrolls older individuals without known dementia, all of whom agreed to annual clinical evaluations and brain donation at death^59^. Both studies were approved by an Institutional Review Board of Rush University Medical Center, and all participants signed informed and repository consents, as well as an Anatomic Gift Act.

The comprehensive dataset used here comprises 172,659 cells, including astrocytes, excitatory neurons, inhibitory neurons, oligodendrocytes, microglia, endothelial cells, and pericytes, which were annotated using unsupervised clustering algorithms in the aforementioned study. The processed single-cell data and corresponding cell type annotations were obtained from Synapse (syn16780177). To mitigate batch effects, the original ROSMAP study adopted a carefully designed pooling strategy, wherein nuclei from multiple participants were profiled together in each batch (typically eight per batch), with samples randomized and balanced for clinical diagnosis, pathological characteristics, and sex^58^. This design was intended to reduce technical variability across batches, and as such, no additional computational batch correction was applied in the original analysis and this work.

This dataset consists of cells annotated by the Seurat pipeline that expressed marker genes from multiple cell types, such as endothelial cells and pericytes, indicating the presence of doublet cells that required further refinement. We employed Azimuth^60^ as an additional reference and retained cells that exhibited consistent cell type annotations from both the Cain et al. study and the Azimuth prediction, thereby ensuring a high level of annotation accuracy and reliability in our subsequent analyses. This step also further minimizes both biological and technical batch effects, including inter-individual and sex-specific variation. The filtered dataset consists of approximately 21,000 cells.

**Fig. 11:**
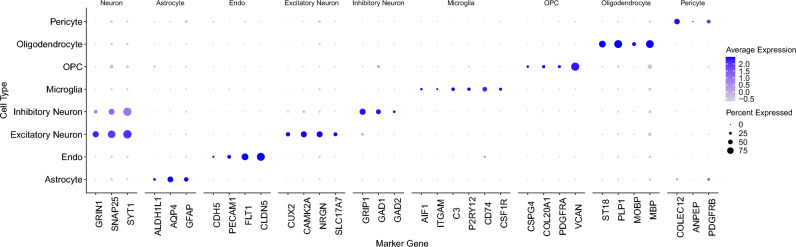
Expression levels of brain cell marker genes across the cell types in the ROSMAP dataset. The size of each dot represents the proportion of cells in a particular cluster or cell type that express a given gene. A larger dot indicates that a higher percentage of cells in that cluster express the gene. The color of each dot reflects the average expression level of the gene in the cells that express it; darker blue indicates higher expression levels.

Figure 11 represents the expression levels of several well-known brain marker genes^11,61,62^ across the cell types assigned in this dataset. One observes that there is a strong correspondence between marker gene expression levels and cell identities, which underscores the reliability of the cell type annotations in this filtered dataset.

Lastly, the mouse primary motor cortex (MOp) dataset analyzed in this study was obtained from the NeMO Analytics portal, based on the comprehensive single-cell transcriptomic and epigenomic atlas published by Yao et al^63^. The original study generated a multimodal molecular census of the adult mouse primary motor cortex by integrating large-scale single-cell RNA sequencing (scRNA-seq), ATAC-seq, and methylation data across diverse neuronal and non-neuronal cell populations. This extensive atlas, encompassing hundreds of thousands of cells, provides a unified hierarchical taxonomy of cortical cell types, ranging from broad neuronal and glial classes to fine-grained subclasses defined by transcriptional, spatial, and electrophysiological characteristics. For the present analysis, we utilized a processed subset of this dataset comprising approximately 10,000 cells and 27,000 genes. The cells are annotated into 17 subclasses representing major excitatory and inhibitory neuronal types as well as glial populations. This selection preserves the key cellular diversity of the MOp region while maintaining a manageable data size for comparative analyses. The dataset was downloaded and preprocessed through the NeMO Analytics interface, ensuring consistency with the standardized pipelines and annotations provided in the original MOp atlas.

### Preprocessing techniques

**Fig. 12:**
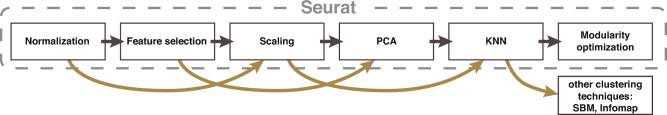
Schematic representation of the methodology. The Seurat pipeline includes all steps up to network clustering via modularity maximization. The alternative pipeline allows for flexibility by enabling exploration of the Seurat pipeline steps, with the option to skip one or more steps until the KNN network is generated (paths shown in yellow). Additionally, the alternative pipeline offers the choice of using clustering methods beyond modularity maximization, such as SBM and Infomap.

#### Preprocessing pipeline using Seurat R package

In the preprocessing pipeline for scRNA-seq data using the Seurat package in R^18^, several essential steps are followed as seen in Fig. 12. Initially, a normalization function NormalizeData() is applied to correct technical biases, such as varying sequencing depths and library sizes, and normalize expression levels across cells, essential for ensuring comparability and reducing technical noise during downstream analysis. Identifying genes that exhibit high variability across cells is crucial for capturing informative features: this is achieved through the FindVariableFeatures() function. Scaling the data with ScaleData() standardizes expression values, preventing genes with larger expressions from dominating subsequent analyses. Dimensionality reduction is then performed using PCA with RunPCA(), extracting the major sources of variation in the dataset and effectively reducing the influence of high-dimensional noise. Finally, FindNeighbors() calculates cell similarities, laying the groundwork for tasks such as clustering and visualization. K-Nearest Neighbors (KNN) and Shared Nearest Neighbors (SNN) algorithms are employed; through KNN, each cell identifies its closest neighbors based on similarity metrics, constructing a weighted undirected network where cells are connected to their K most similar counterparts. Additionally, SNN evaluates shared neighbors between cells, emphasizing connections that go beyond immediate proximity and capture higher-order relationships. By leveraging these network-based approaches, this technique transforms the single-cell expression data into a network representation that encapsulates complex cellular interactions and structural properties. This network is then used as an input for the clustering analysis, which partition cells into biologically meaningful groups based on their network connectivity patterns.

#### Alternative preprocessing pipeline

The process of constructing biological networks from diverse datasets is a complex task as it encompasses a wide range of methodologies and critical decisions. These choices can significantly influence the network representation and, as a consequence, they impact cluster detection. In functional brain networks, one common approach involves the calculation of correlation coefficients (either Pearson or Spearman) across the time series of functional Magnetic Resonance Imaging (fMRI) or electroencephalogram (EEG) signals^64^. This method facilitates the identification of synchronous activity patterns among brain regions, where nodes represent distinct areas of the brain. Similarly, gene co-expression networks are derived by calculating the correlation between transcriptomes for each gene pair across a population of individuals. In these networks, genes are represented as nodes, typically numbering between 10,000 to 20,000.

A common preprocessing pipeline may involve several steps, such as transformation or normalization^65,66^, feature selection^60^, scaling and dimension reduction^67^. The normalization ensures that the gene expression values are comparable across cells, and can be done by dividing the counts for each cell by the total count. In addition, the data can be log-transformed (using $\log (\textrm{count}+1)$), which reduces the effects of outliers and stabilizes the variance, thereby mitigating technical noise. Data can then be centered and scaled through standardization, which involves computing the z-scores of the values based on the mean and standard deviation of the null model distribution. In this case, the null model distribution is derived from an estimated mean-variance relationship, obtained by fitting a second-degree polynomial to the variance-vs-mean curve across all gene entries. The standardization procedure adjusts each gene entry by subtracting its mean and dividing by the estimated standard deviation derived from the polynomial fit. Next, features are selected to reduce noise in the data and better encode genes that capture significant biological variability.

Since the number of genes in scRNA-seq can be large, and many gene profiles may correlate to each other, dimension reduction can be used to reduce the computational cost of network construction, while maintaining the relevant relationships between cells. A common approach is PCA^67^, which reduces the data to linear combinations of maximum variance.

The network of cells can be constructed through several approaches. The most common is computing the similarity between all pairs of cells. However such a procedure does not scale well with the size of the networks. Alternatively, one can use KNN^65^; both approaches use Pearson’s correlation between the cell profiles across genes. It is generally unfeasible to calculate, store, and analyze all pairwise similarities, particularly for large datasets. For that reason, sparsification strategies are normally employed, like removing all similarities lower than a fixed threshold, or using localized methods, such as the disparity filter^68^. For very large datasets, neither of these options may be suitable given their computational costs and required memory to run. In that case, approximated approaches for KNN can be used, such as Nearest Neighbor Descent^69^.

While all the aforementioned steps of the preprocessing pipeline have been used in most procedures for network generation, it is unclear how they affect the network structure and, consequently, the detected clusters. Here, we investigate how different choices for the pipeline demonstrated in Fig. 12 may lead to different clustering performances.

**Fig. 13:**
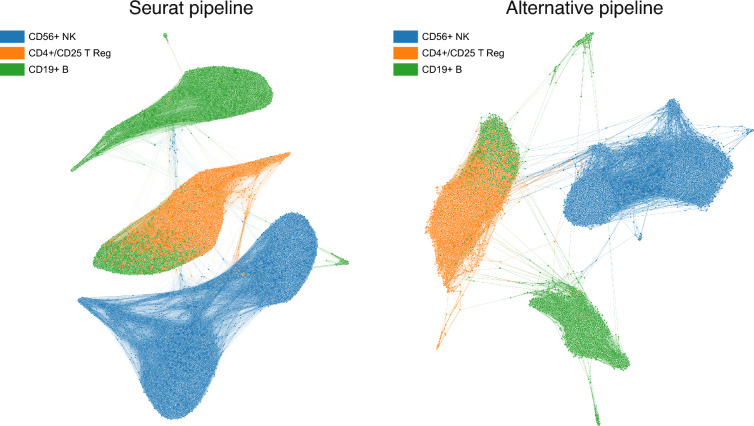
PBMC 20k network: Illustration of the networks obtained from 20k simple PBMC dataset. These networks are generated using Seurat and the alternative pipelines.

**Fig. 14:**
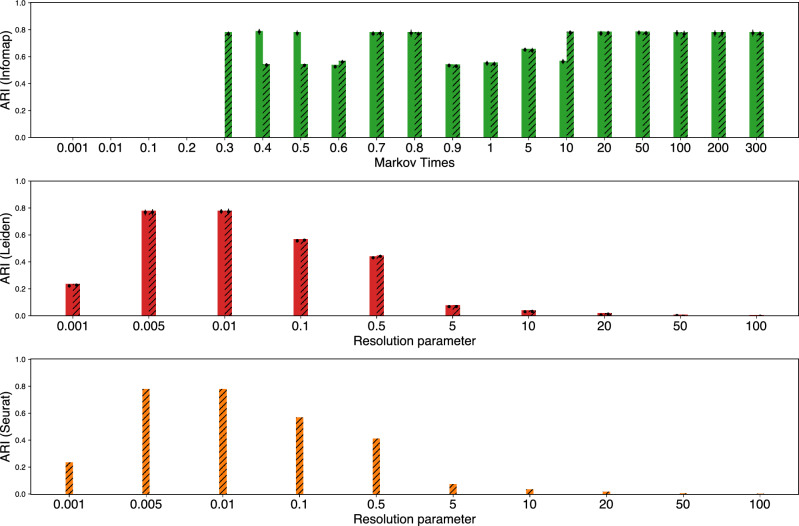
ARI vs tuning parameter - PBMC 20k dataset: ARI across different resolution parameters for the network generated from 20k simple PBMC dataset.

**Fig. 15:**
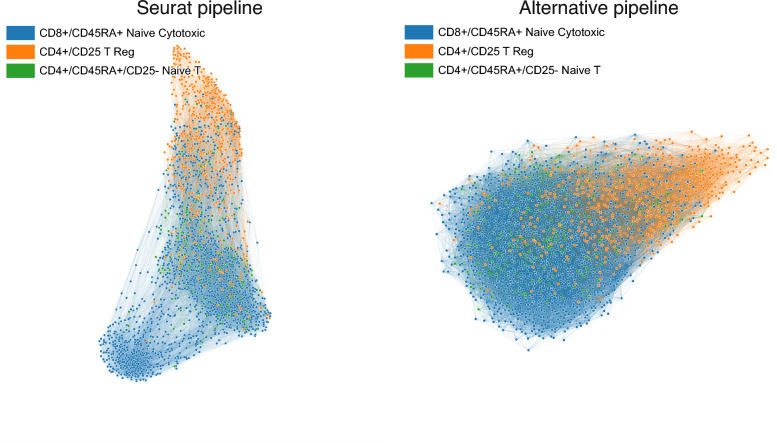
PBMC 2k network: Illustration of the networks obtained from the 2k challenging PBMC dataset. These networks are generated using Seurat and the alternative pipelines.

**Fig. 16:**
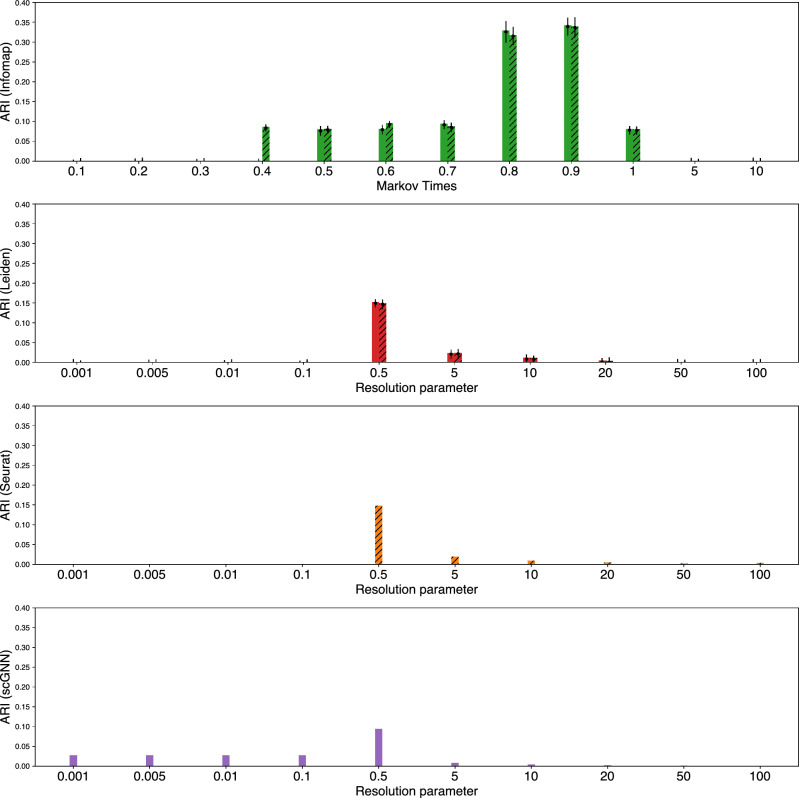
ARI vs tuning parameter - PBMC 2k dataset: ARI across different resolution parameters for the network generated from the 2k challenging PBMC dataset.

**Fig. 17:**
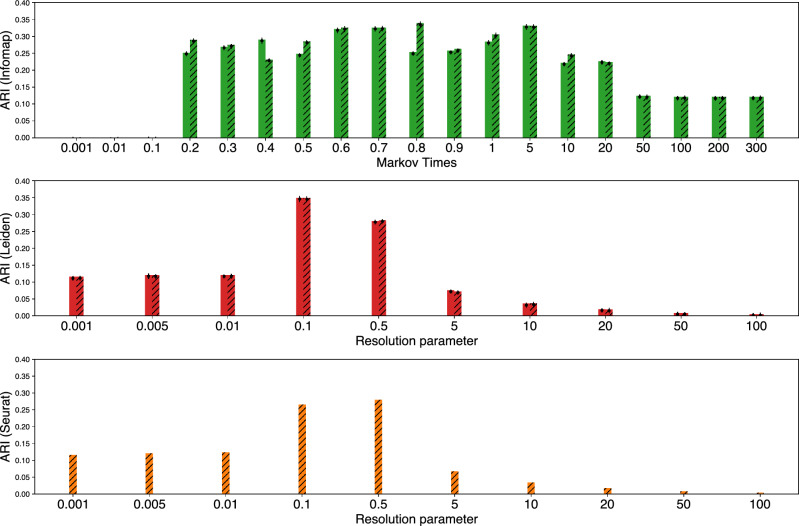
ARI vs tuning parameter - PBMC full dataset: ARI across different resolution parameters or Markov times for the Seurat-generated network from the 68k PBMC full dataset.

**Fig. 18:**
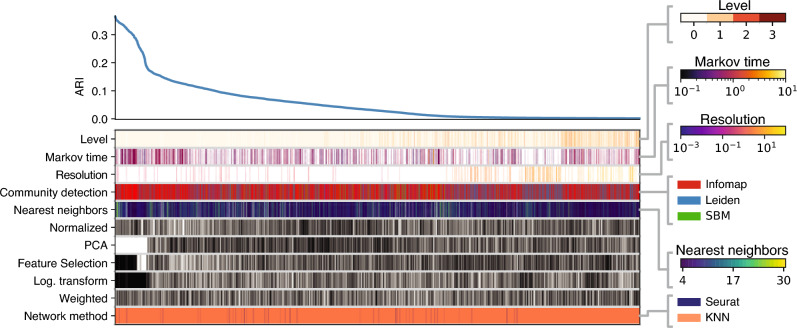
ARI across different configurations of alternative preprocessing pipelines for the 2k PBMC challenging dataset. Same as figure 5 but for the top 5000 procedures.

**Fig. 19:**
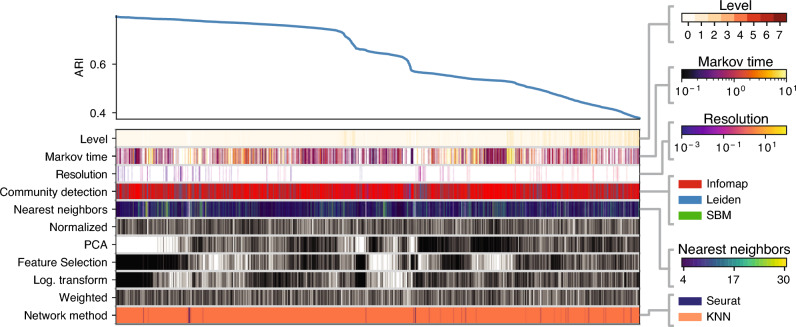
ARI across different configurations of alternative preprocessing pipelines for the 68k PBMC full dataset. Same as figure 6 but for the top 5000 procedures.

**Fig. 20:**
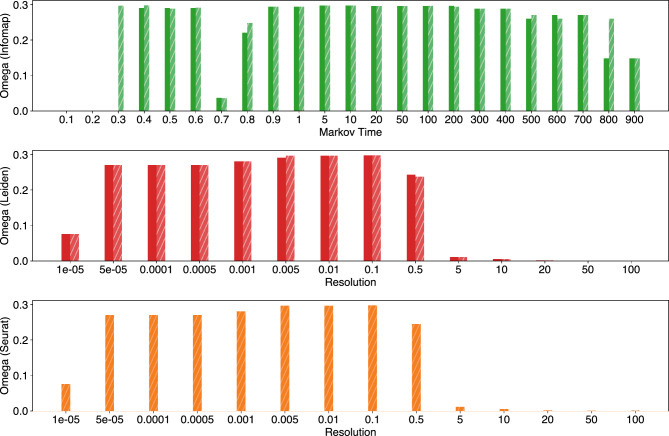
Omega index vs tuning parameter - ROSMAP dataset: Omega index across different resolution parameters or Markov times for the Seurat-generated network from ROSMAP full dataset.

## Figures and Tables

**Fig. 21:**
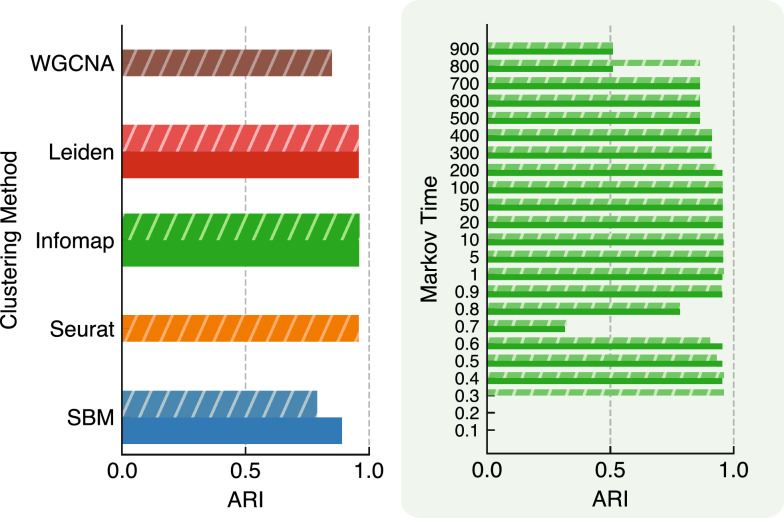
ARI obtained using different methods in ROSMAP: Adjusted Rand Index (ARI) between cell types and detected clusters for SBM, Seurat, Infomap, Leiden, and WGCNA in the ROSMAP full dataset. Both the weighted and unweighted versions of the same networks were considered for algorithms that can handle both. The zoomed-in panel illustrates the ARI across different Markov times using Infomap.

**Fig. 22:**
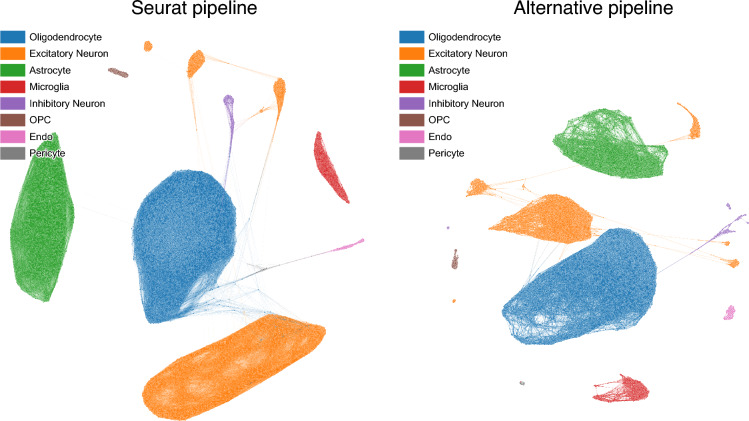
ROSMAP network: Illustration of the networks obtained from ROSMAP dataset. These networks are generated using Seurat and the alternative pipelines.

**Fig. 23:**
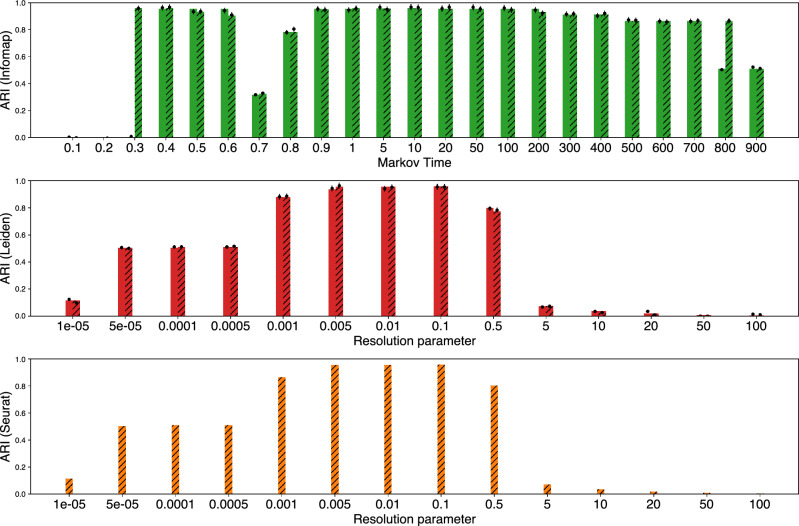
ARI vs tuning parameter - ROSMAP: ARI across different resolution parameters for the network generated from the ROSMAP dataset.

**Fig. 24:**
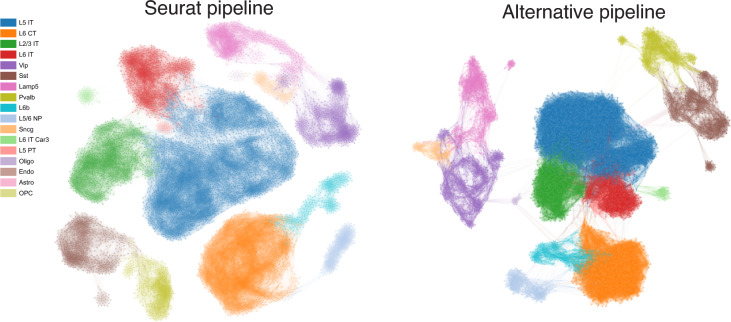
MOp network: Illustration of the networks obtained from the MOp dataset. These networks are generated using Seurat and the alternative pipelines.

**Fig. 25:**
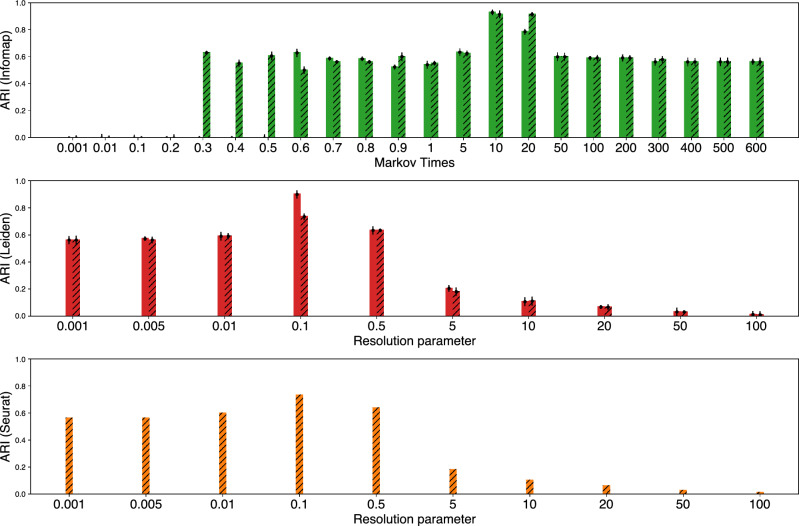
ARI vs tuning parameter - MOp: ARI across different resolution parameters for the network generated from the MOp dataset.

**Fig. 26:**
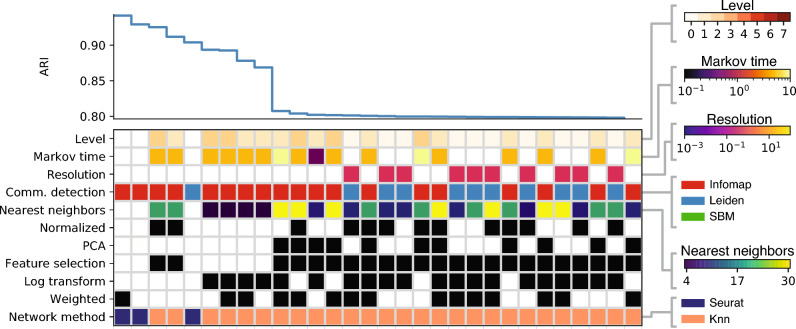
ARI across different configurations of alternative preprocessing pipelines for the MOp dataset. Same as Fig. 10 but for the top 5000 procedures.

## Data Availability

This study is based entirely on publicly available datasets. The ROSMAP single-nucleus RNA-seq (snRNA-seq) data are available from the AD Knowledge Portal at https://www.synapse.org/#!Synapse:syn16780177. Processed data can be explored via a Shiny app at https://vmenon.shinyapps.io/rosmap_snrnaseq24/. The bulk RNA-seq dataset is available at https://www.synapse.org/#!Synapse:syn3388564. The ROSMAP proteomics dataset can be accessed via https://www.synapse.org/#!Synapse:syn17015098. Access to these datasets is granted for general research use in accordance with the AD Knowledge Portal’s data access and attribution requirements: https://adknowledgeportal.synapse.org/#/DataAccess/Instructions. The PBMC 68k single-cell RNA-seq dataset is available from the NCBI Short Read Archive under accession number SRP073767, and can also be accessed at https://support.10xgenomics.com/single-cell/datasets. The code to reproduce the results in this work is available at https://github.com/FatemehFN/cell_type_differentiation.

## Appendix

The Omega Index $\Omega (s_1, s_2)$ measures the similarity between two clustering solutions that may include overlapping clusters. To calculate it, we first determine the observed agreement, $\text {Obs}(s_1, s_2)$, by summing the proportion of pairs that both clustering solutions agree to assign to the same number of clusters. This is expressed as

$$\text {Obs}(s_1, s_2) = \sum _{j=0}^{\min (J,K)} \frac{A_j}{N}$$

where $A_j$ is the count of pairs that both solutions agree to assign to $j$ clusters, and $N$ is the total number of pairs. We then calculate the expected agreement, $\text {Exp}(s_1, s_2)$, as

$$\text {Exp}(s_1, s_2) = \sum _{j=0}^{\min (J,K)} \frac{N_{j1} \cdot N_{j2}}{N^2}$$

where $N_{j1}$ and $N_{j2}$ represent the total pairs assigned to $j$ clusters in solutions 1 and 2, respectively. Finally, the Omega Index is calculated as

$$\Omega (s_1, s_2) = \frac{\text {Obs}(s_1, s_2) - \text {Exp}(s_1, s_2)}{1 - \text {Exp}(s_1, s_2)}$$

This index ranges from 0 to 1, with 1 indicating perfect agreement between the clustering solutions.
